# Supplementary figures and images for: Early warning signals in motion inference
Source: PLoS Comput Biol. 2020 May 29;16(5):e1007821. doi: 10.1371/journal.pcbi.1007821 (PMC7259514; doi:10.1371/journal.pcbi.1007821)

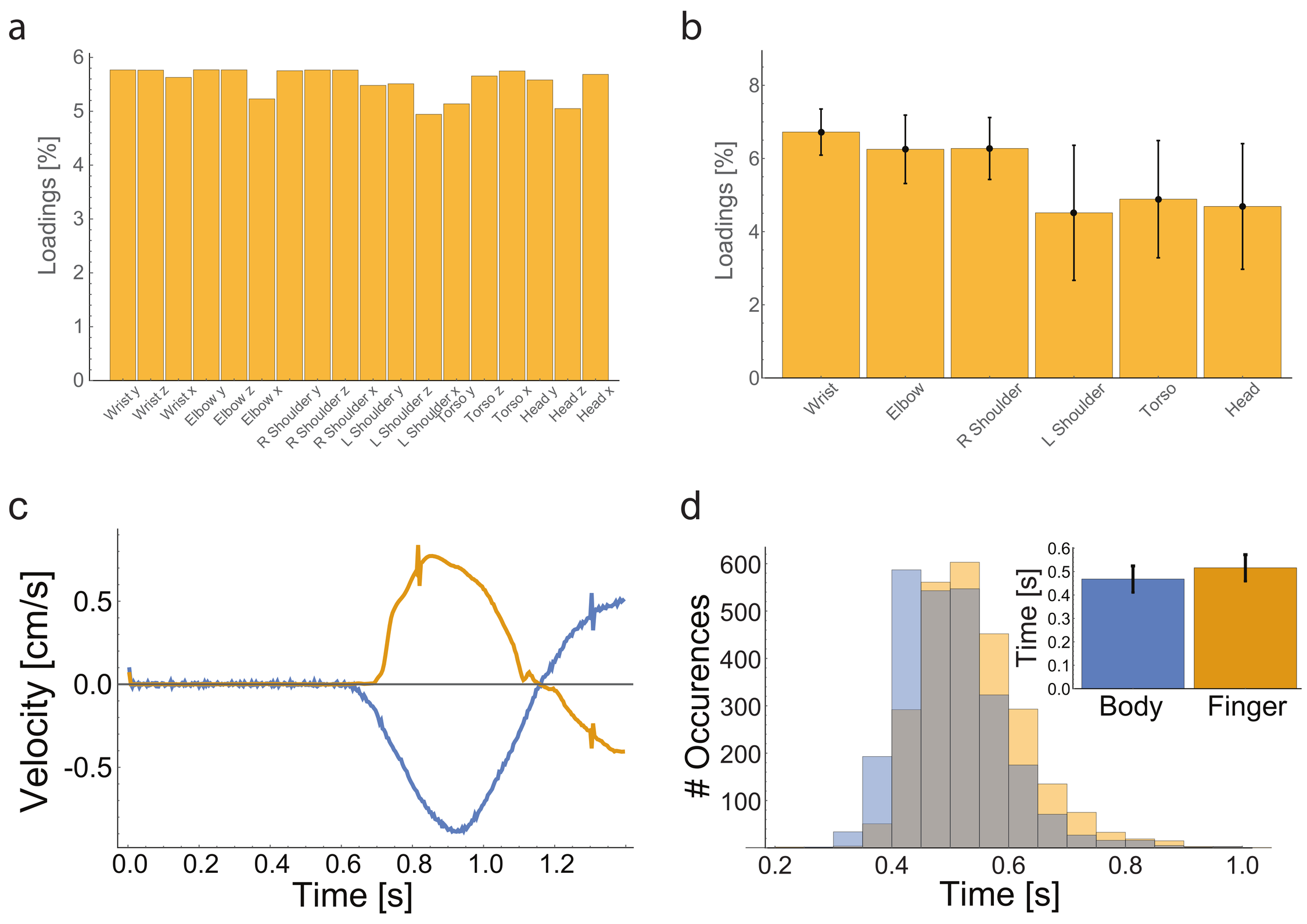

Supplement: S1 Fig — (TIF) [file pcbi.1007821.s001.tif]

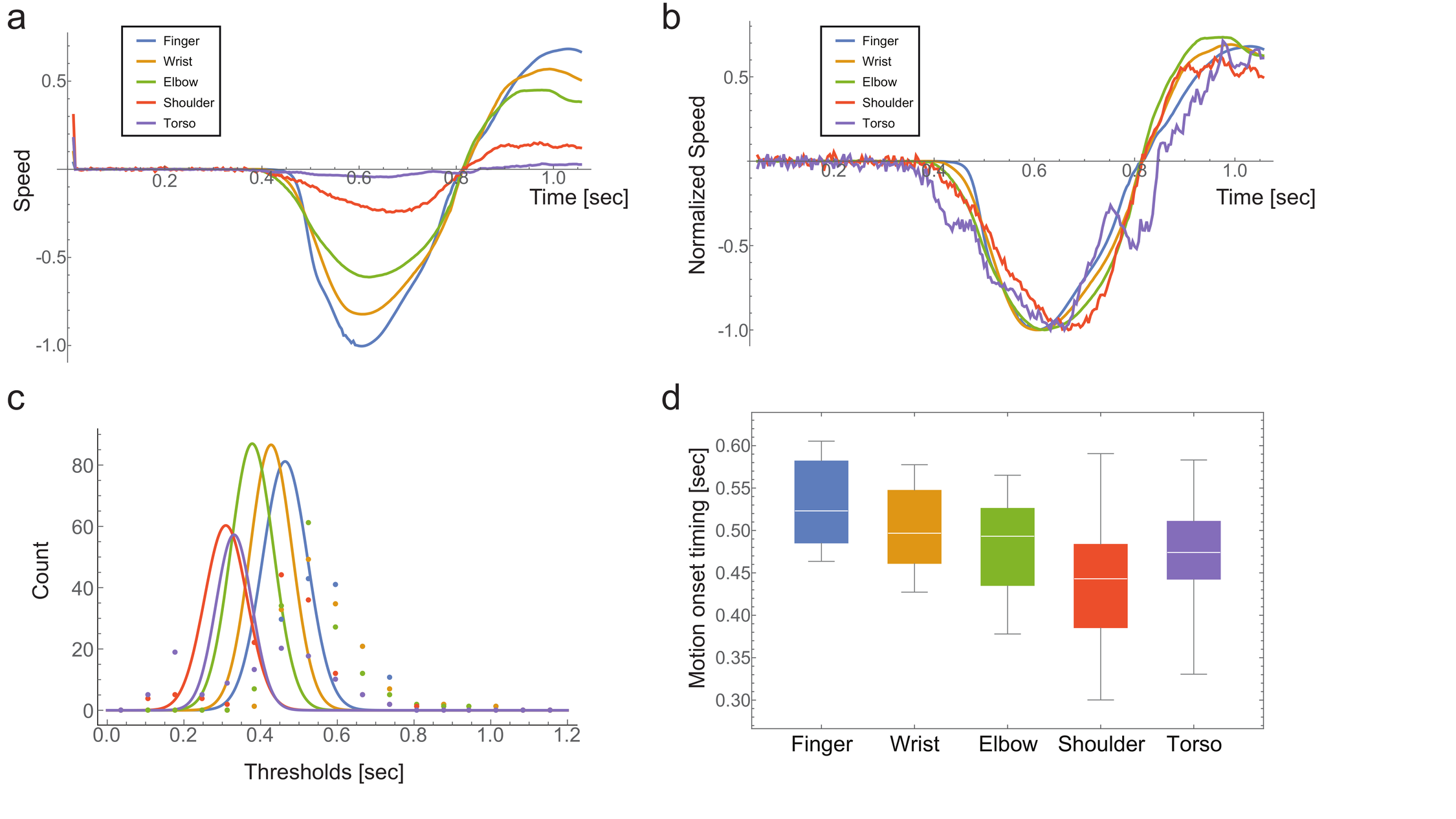

Supplement: S2 Fig — (TIF) [file pcbi.1007821.s002.tif]

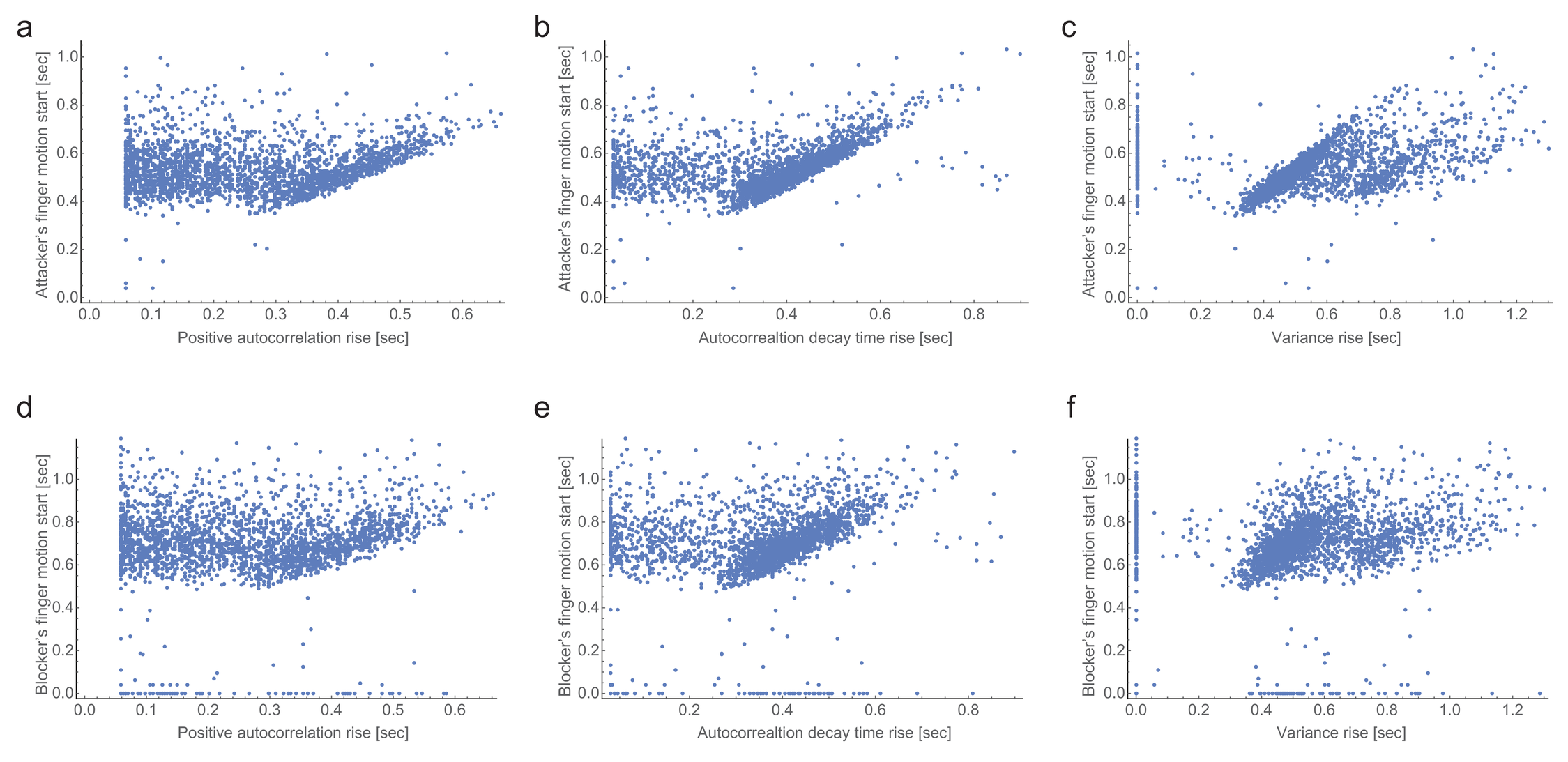

Supplement: S3 Fig — (TIF) [file pcbi.1007821.s003.tif]

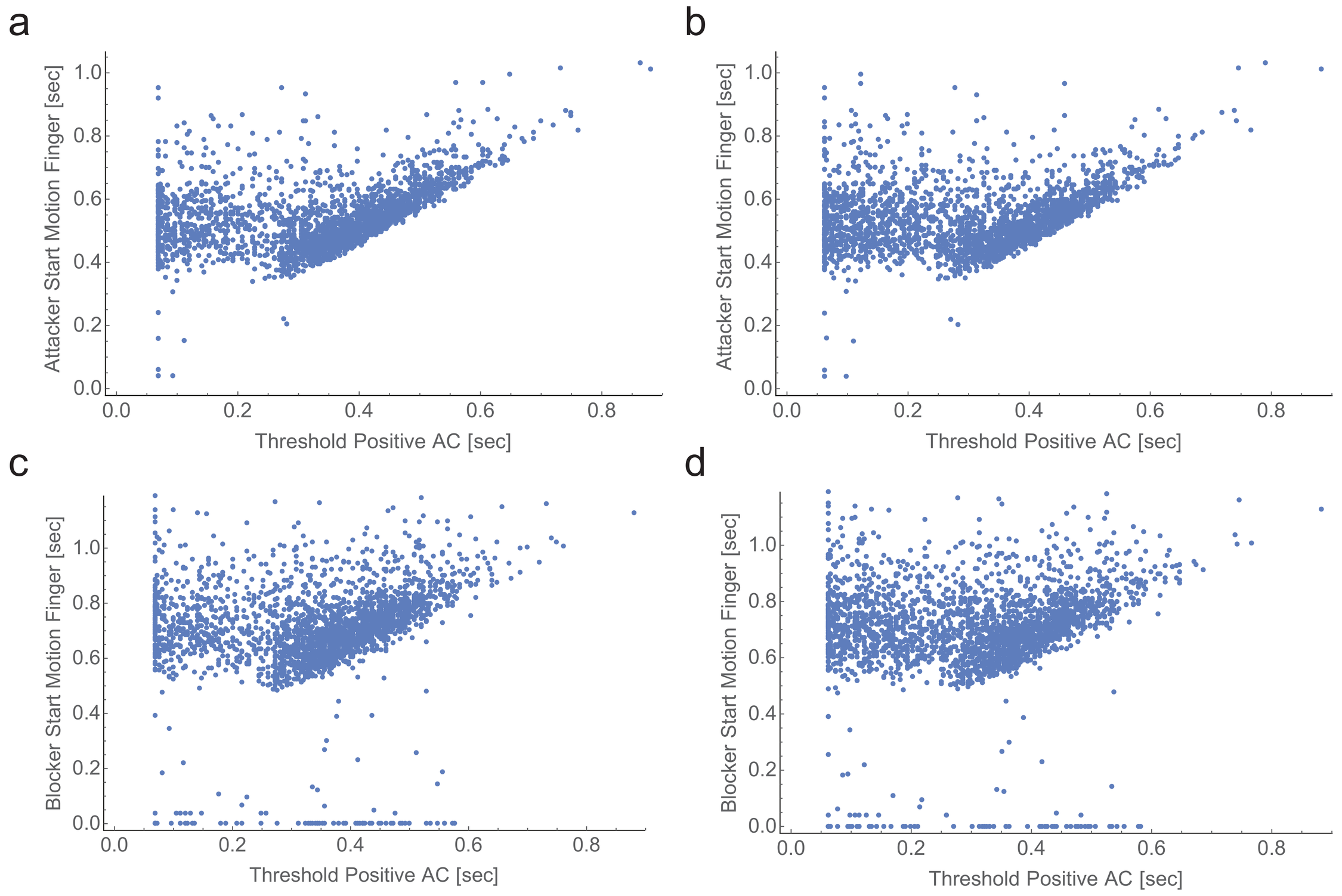

Supplement: S4 Fig — (TIF) [file pcbi.1007821.s004.tif]

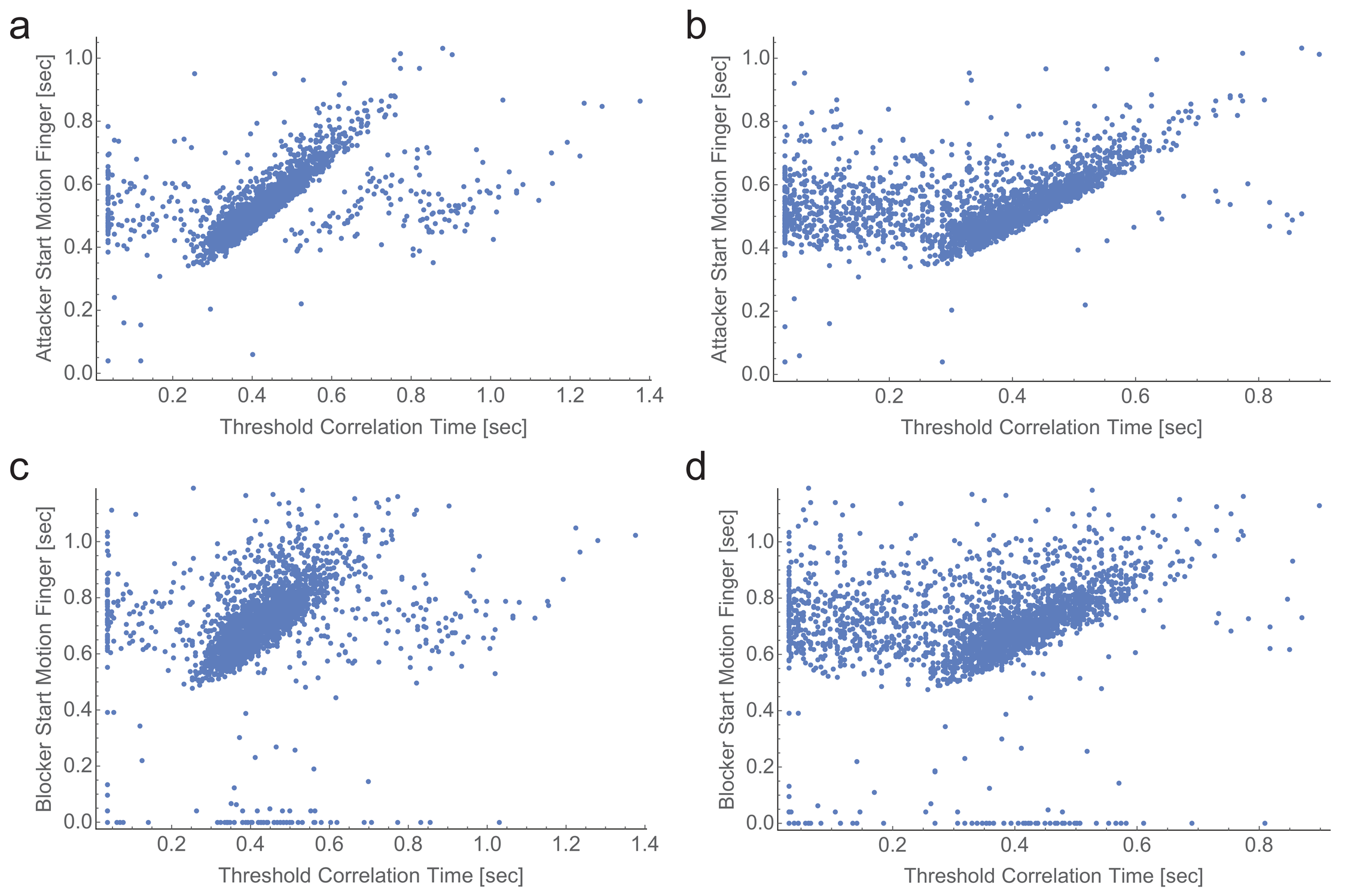

Supplement: S5 Fig — (TIF) [file pcbi.1007821.s005.tif]

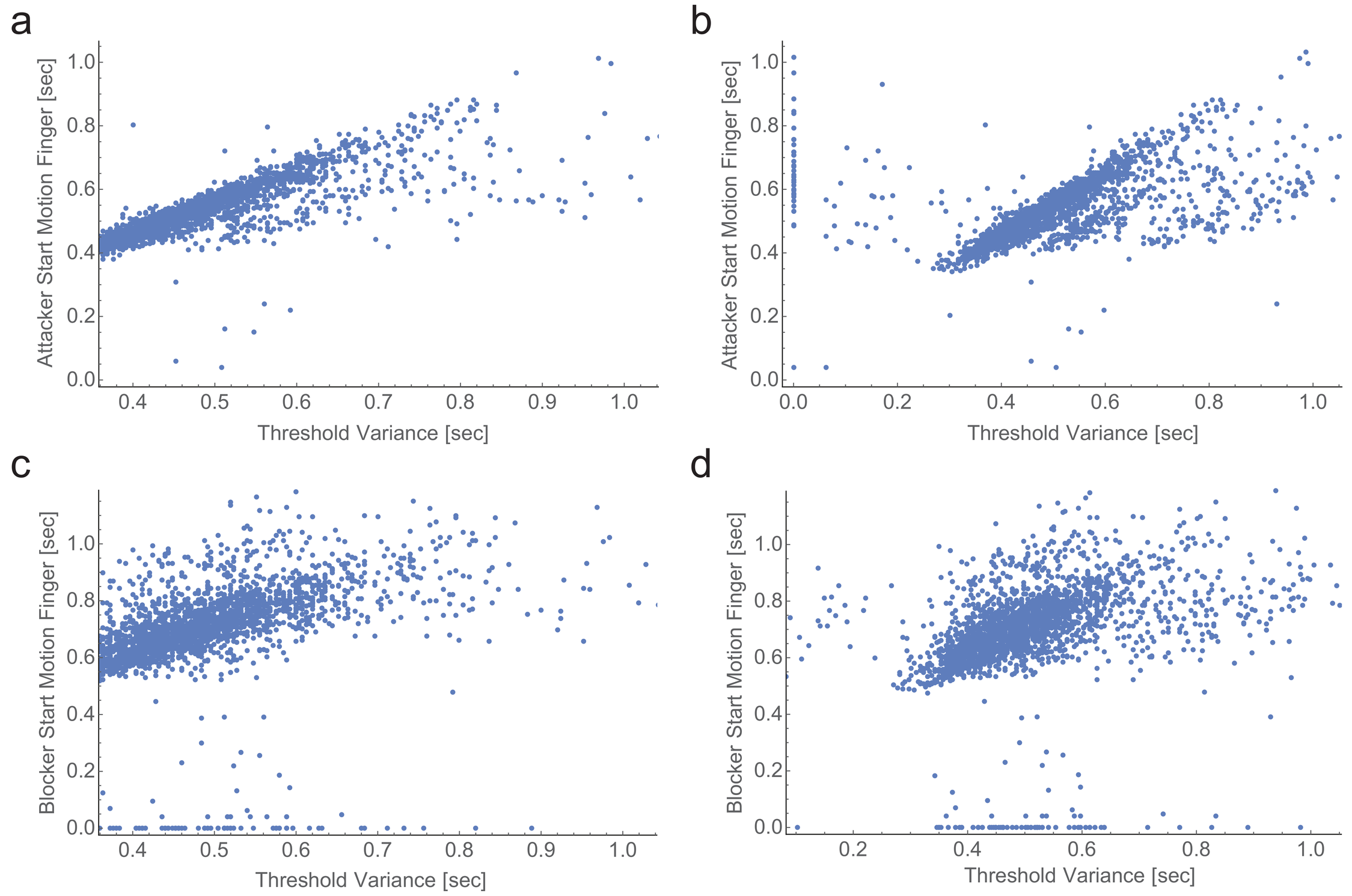

Supplement: S6 Fig — (TIF) [file pcbi.1007821.s006.tif]

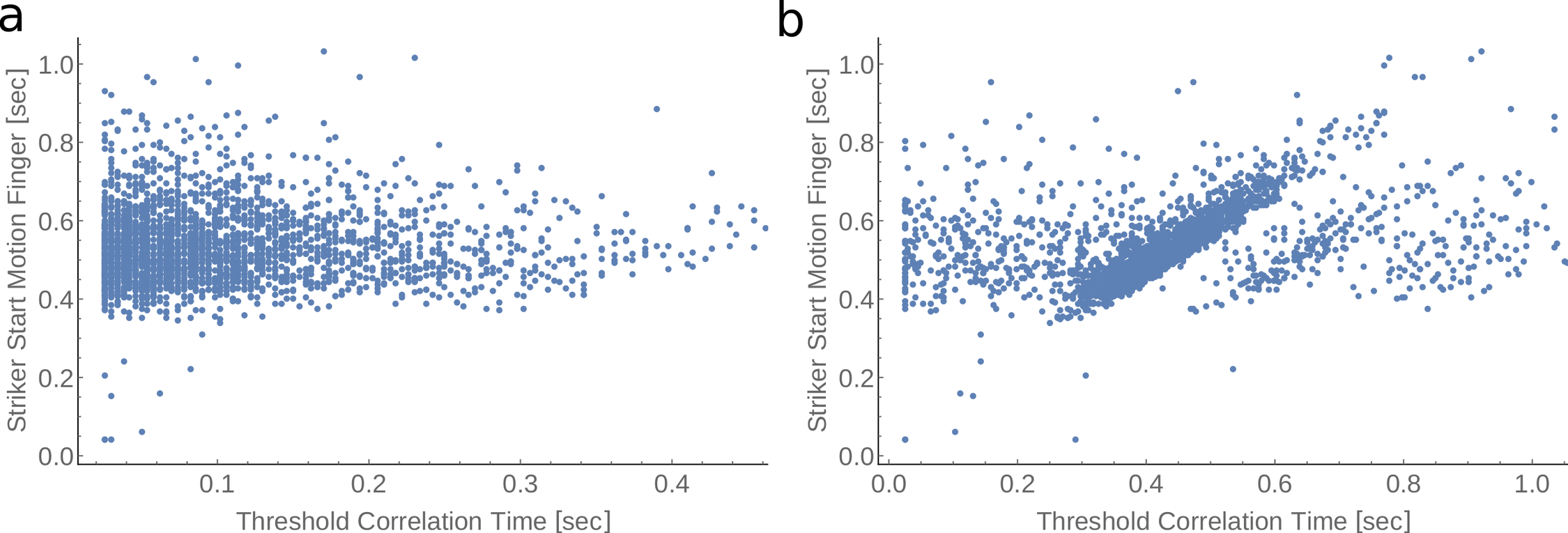

Supplement: S7 Fig — (TIF) [file pcbi.1007821.s007.tif]

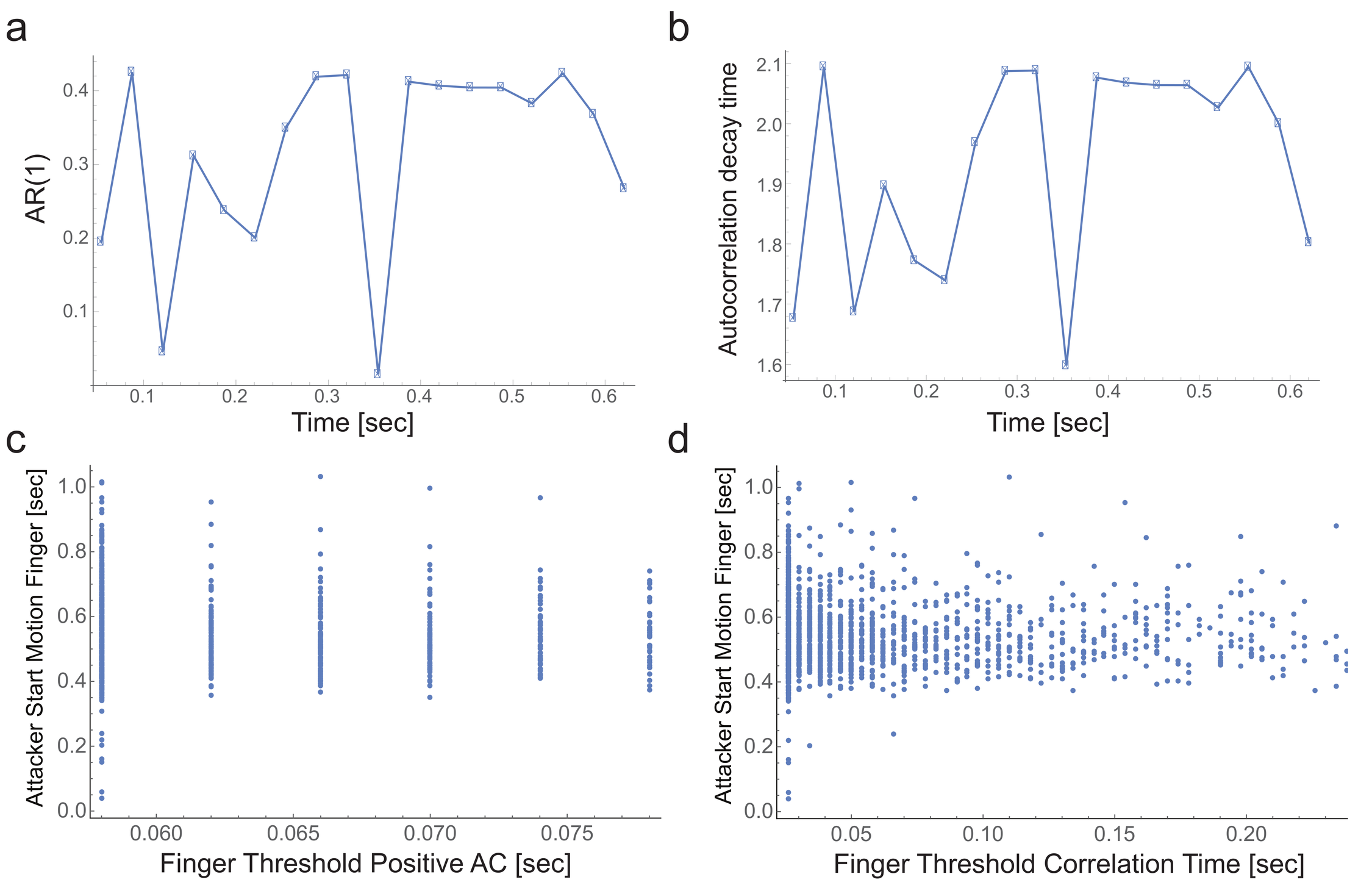

Supplement: S8 Fig — (TIF) [file pcbi.1007821.s008.tif]

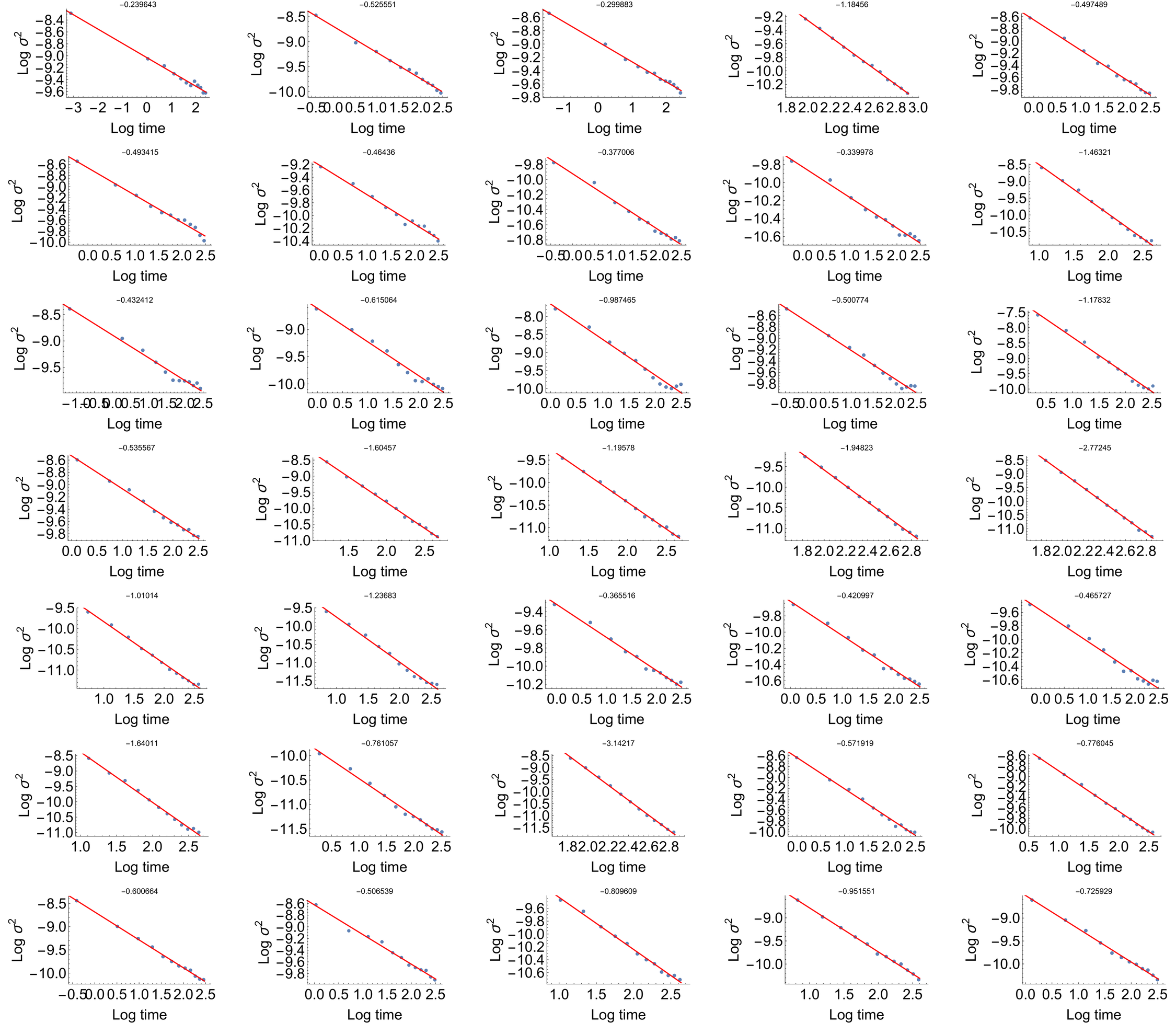

Supplement: S9 Fig — (TIF) [file pcbi.1007821.s009.tif]

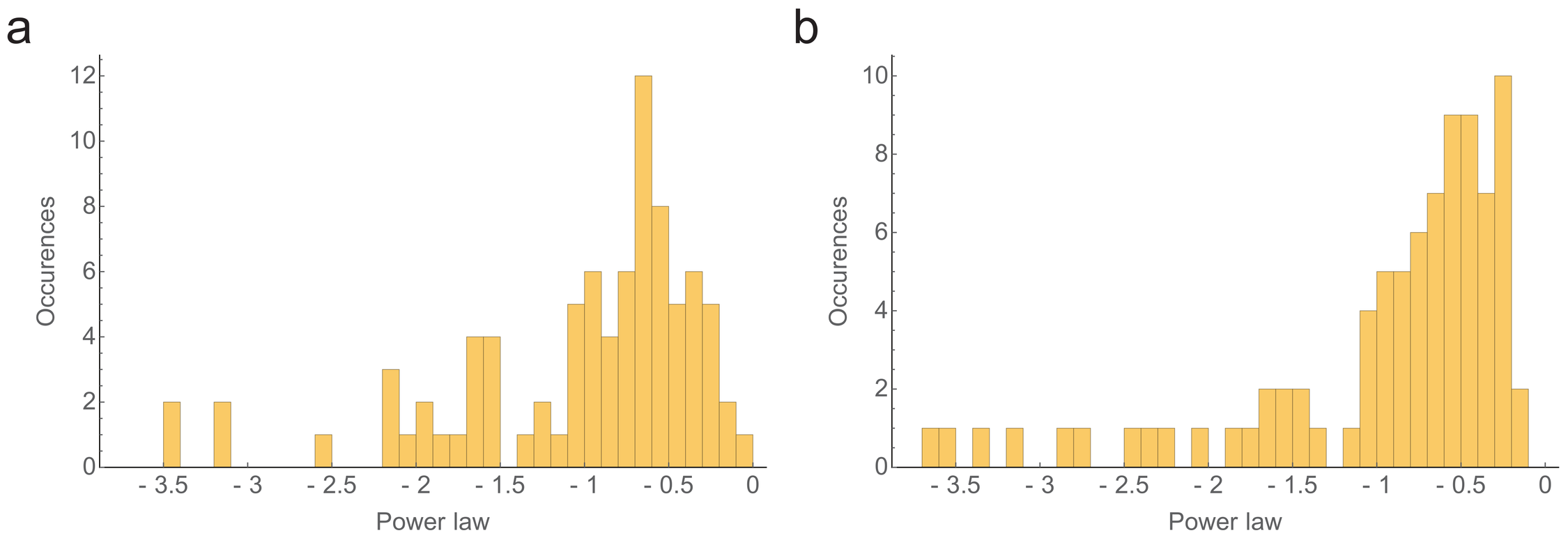

Supplement: S10 Fig — (TIF) [file pcbi.1007821.s010.tif]

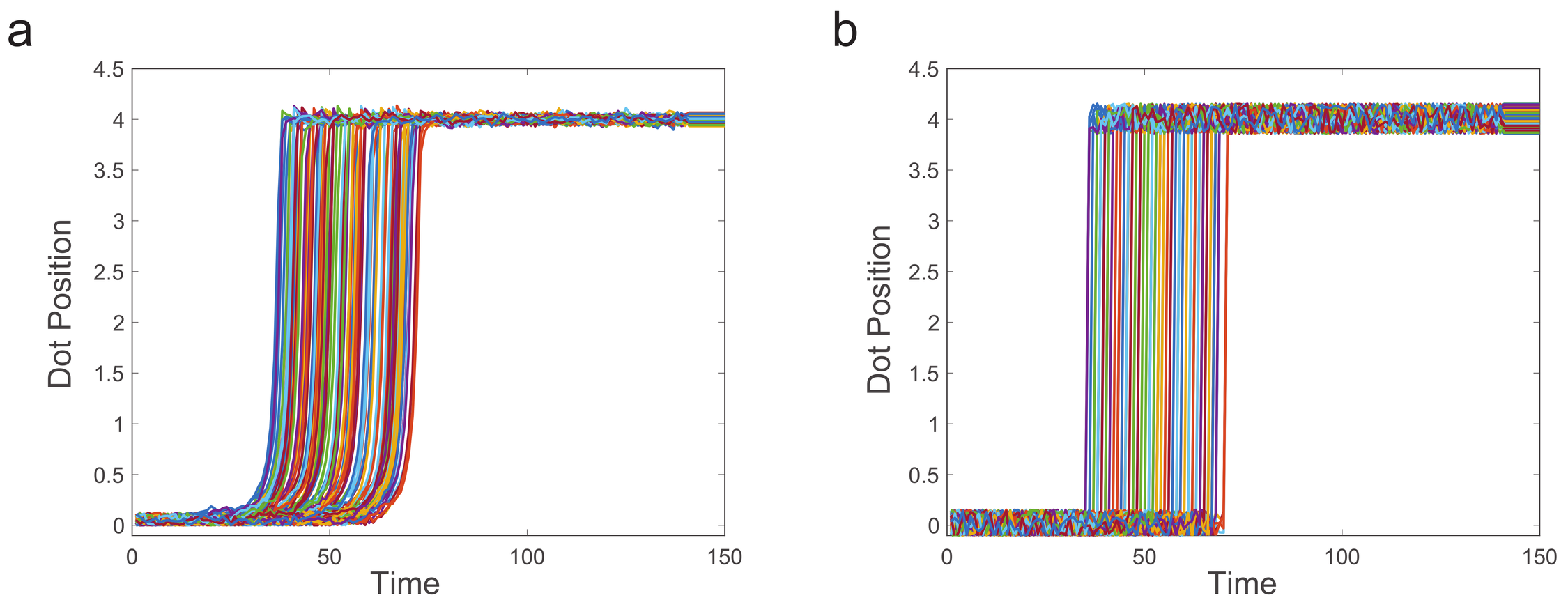

Supplement: S11 Fig — (TIF) [file pcbi.1007821.s011.tif]

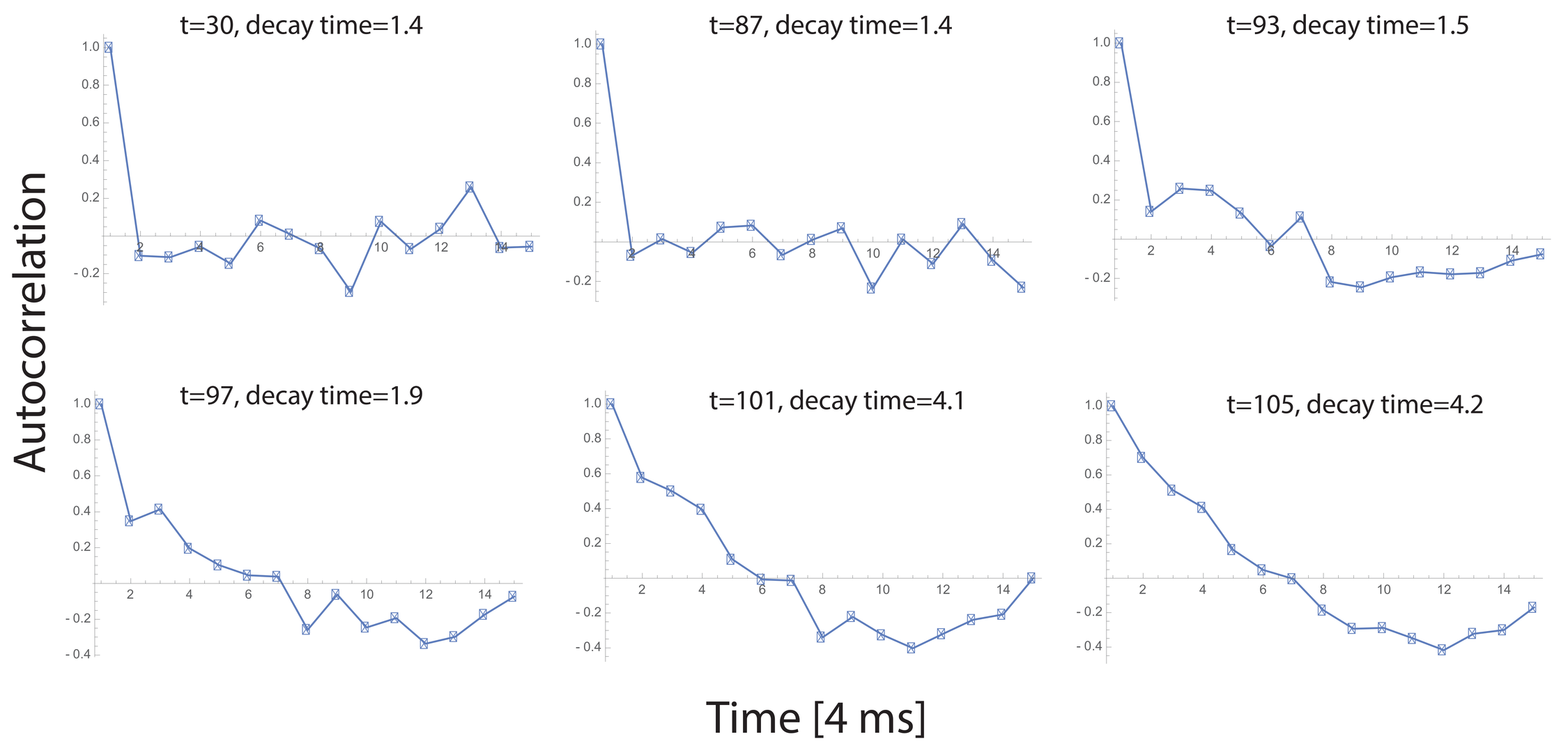

Supplement: S12 Fig — (TIF) [file pcbi.1007821.s012.tif]

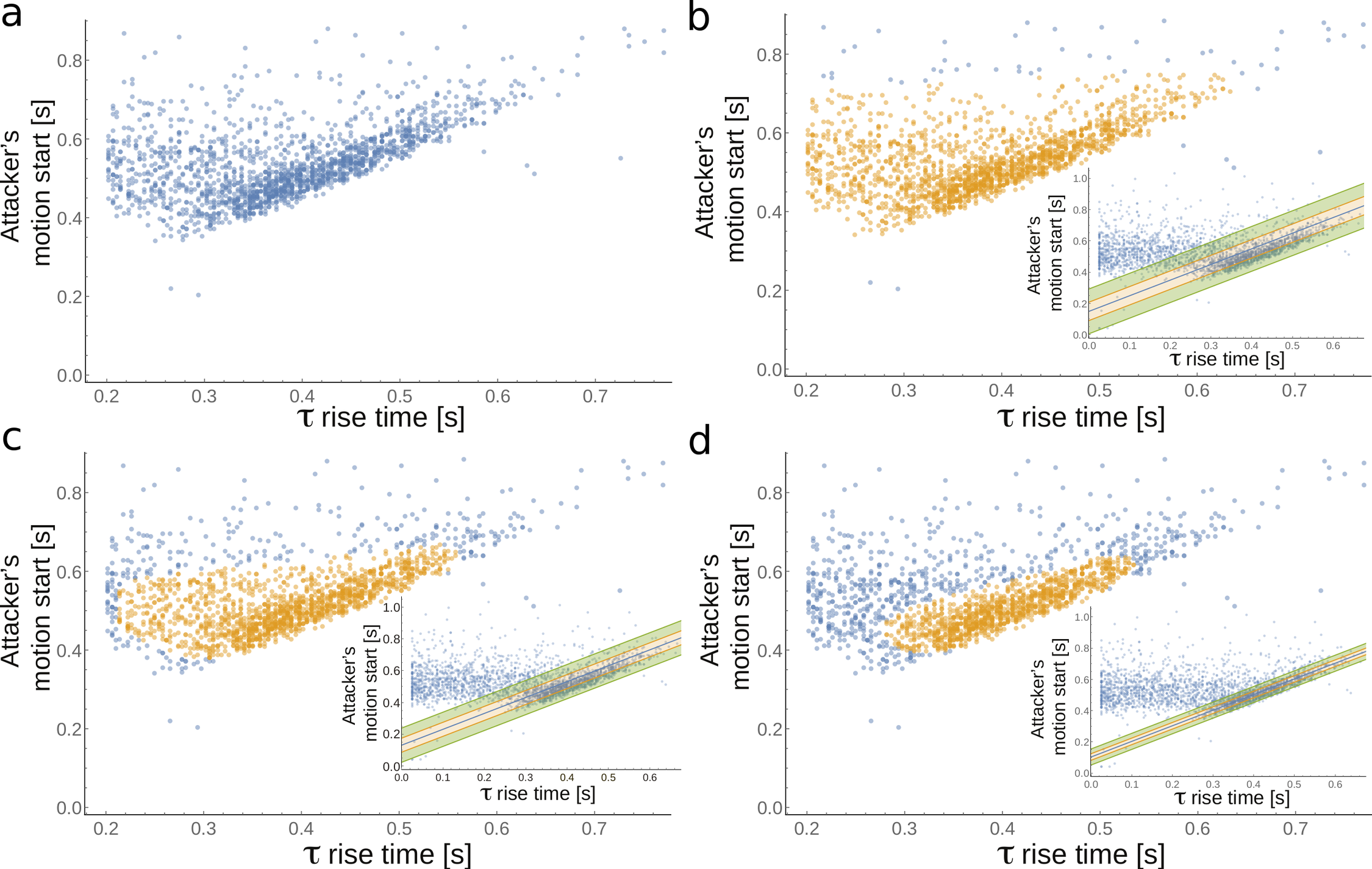

Supplement: S13 Fig — (TIF) [file pcbi.1007821.s013.tif]
