# Supplementary material for: Early warning signals in motion inference
Source: PLoS Comput Biol. 2020 May 29;16(5):e1007821. doi: 10.1371/journal.pcbi.1007821 (PMC7259514; doi:10.1371/journal.pcbi.1007821)
Supplement: S1 Text — (PDF) [file pcbi.1007821.s014.pdf]

Early warning signals in motion inference - Supporting Information

Yuval Hart, Maryam Vaziri-Pashkam, and L. Mahadevan

Contents

S1 First principal component of Attackers' motion is its center of mass **1**

S2 Attackers' motion starts from the center of the body to its periphery **2**

S3 Different window sizes show similar timing for the early warning signals **2**

S4 Autocorrelation decay time threshold balances accuarate detection and false positives **3**

S5 Finger dot motion does not show early warning signals **4**

S6 The divergence of motion variance near the critical transition point is of a fold-bifurcation type **5**

S7 Variance divergence power-law is independent of the window size **5**

S8 Simulations of fold-bifurcation critical transition **5**

S9 Individual response times show a significant difference between the fold-bifurcation and uncorrelated noise motion simulations **6**

S10 Screening of outliers in the relation of motion onset to the early warning signals **6**

SI Figures **8**

SI Tables **19**

S1 First principal component of Attackers' motion is its center of mass

In order to reveal the motion cues that enable Blockers' fast response, we analyzed the principal components (PC) of Attackers' motion. We used the 6 sensors connected to Attackers head, torso, right and left shoulders, elbow, and wrist (each conveys a 3d coordinate position along each trial). For each trial out of the 2550 Attacker-Blocker dynamics, we computed the first PC of motion. We find that the first PC accounts for 99% of the total variance and reflects an equal contribution from each of the sensors (mean loadings of each sensor's coordinate and 95% CIs: Wrist=6.7%, 95% CI=[5.8,8.1], Elbow=6.3%, 95% CI=[4.2,7.8], Right Shoulder=6.3%, 95% CI=[4.1,7.6], Left Shoulder=4.5%, 95% CI=[0.4,6.9], Torso=4.9%, 95% CI=[0.7,6.8], Head=4.7%, 95% CI=[0.5,6.8], Fig S1a,b). Fig S1 further shows that body motion precedes finger motion by an average of about 50ms, thus not explaining the early warning signals observed in the Attacker's motion fluctuations (Fig S1c,d).

S2 Attackers' motion starts from the center of the body to its periphery

To assess the contribution of different body parts to the onset of motion, we calculated for each body sensor (torso, right shoulder, elbow, wrist, and finger) its speed along the trial (Fig S2a). For each sensor, we mark the onset of motion at the time its speed reaches 10% of its maximal value in this trial (since different body sensors display different speeds, we normalized the speeds by their maximal value in the trial, Fig S2b). To overcome outliers in the calculation, we fitted for each Attacker the distribution of timings to a Gaussian (with mean, variance and amplitude as fitting parameters, see Fig S2c). We then aggregated across Attackers the mean values of the Gaussians for each sensor. In the process, we removed two Gaussian fits that showed high variance in their estimate, which indicated a noisy estimate of the Gaussian's mean. We then calculated the median timing for each sensor. Our results show that motion onset cascades from the center of body (shoulder, torso) to peripheral body parts (elbow, wrist, finger), where shoulder motion onset precedes finger motion onset by 74 ms (median timing for motion onset and 50% CI: Finger: 523 ms, 50% CI=[485,582] ms, Wrist: 497 ms, 50% CI=[461,547] ms, Elbow: 493 ms, 50% CI=[435,526] ms, Shoulder: 443 ms, 50% CI=[385,484] ms, Torso: 474 ms, 50% CI=[442,510] ms, Fig S2d).

S3 Different window sizes show similar timing for the early warning signals

In the main text, we presented the linear regression results of the Attackers' and Blockers' motion onset with the early warning signals, calculated along a window size of 40 ms (Fig S3). In this section, we present the linear regression results of the Attackers' and Blockers' motion onset timings with the early warning signals calculated with a window size of 48 ms and 60 ms.

For the AR(1) early warning signal, a linear regression between the AR(1) sharp rise timing and Attackers' start time of finger motion indicates a high correlation with an average time difference of 132 ms (60 ms window: Δt mean $\pm$ ste =132 $\pm$ 1 ms, $p < 10^{-5}$, Fig S4a) and 137 ms (48 ms window: Δt mean $\pm$ ste = 137 $\pm$ 2 ms, $p < 10^{-5}$, Fig S4b). Similarly, a linear regression between the AR(1) sharp rise timing and the Blockers' start time of finger motion indicates a high correlation with an average time difference of 357 ms (60 ms window: Δt mean $\pm$ ste =357 $\pm$ 47 ms, $p < 10^{-5}$, Fig S4c) and 388 ms (48 ms window: Δt mean $\pm$ ste = 388 $\pm$ 56 ms, $p < 10^{-5}$, Fig S4d).

For the autocorrelation decay time, a linear regression between the timing of autocorrelation decay time sharp rise and Attackers' start time of finger motion indicates a high correlation with an average time difference of 107 ms (60 ms window: Δt mean $\pm$ ste =107 $\pm$ 1 ms, $p < 10^{-5}$, Fig S5a) and 126 ms (48 ms window: Δt mean $\pm$ ste = 126 $\pm$ 1 ms, $p < 10^{-5}$, Fig S5b). Similarly, a linear regression between the timing of autocorrelation decay time sharp rise and the Blockers' start time of finger motion indicates a good correlation (albeit much noisier compared to all other correlations) with an average time difference of 90 ms (60 ms window: Δt mean $\pm$ ste =90 $\pm$ 27 ms, $p = 0.001$, Fig S5c) and 320 ms (48 ms window: Δt mean $\pm$ ste = 320 $\pm$ 37 ms, $p < 10^{-5}$, Fig S5d).

Lastly, for the variance, a linear regression between variance rise and the Attackers' start time of finger motion indicates a high correlation with an average time difference of 50 ms (60 ms window: Δt mean $\pm$ ste =50 $\pm$ 1 ms, $p < 10^{-5}$, Fig S6a) and 23 ms (48 ms window: Δt mean $\pm$ ste = 23 $\pm$ 2 ms, $p < 10^{-5}$, Fig S6b). Similarly, a linear regression between the timing of variance rise and the Blockers' start time of finger motion indicates a high correlation with an average time difference of 234 ms (60 ms window: Δt mean $\pm$ ste = 234 $\pm$ 1 ms, $p < 10^{-5}$, Fig S6c) and 215 ms (48 ms window: Δt mean $\pm$ ste = 215 $\pm$ 2 ms, $p < 10^{-5}$, Fig S6d).

S4 Autocorrelation decay time threshold balances accuarate detection and false positives

In the main text we show the correlation between Attacker's motion onset and Blocker's motion onset with the time of the sharp rise of the autocorrelation decay time signal. We chose the threshold value of increase in the decay time to be set at 20%. Here, we show that this value balances well between the

need for early detection of the rise in the autocorrelation decay time and the need to avoid false detection of events due to a noisy signal. Shown in Fig S7 are the relations between Attacker's motion onset and the time of rise of the autocorrelation decay time for threshold values of 10% and 30%. As seen in the figure, for the threshold value of 10%, there are many events of false positives which mask the relation between the rise in the decay time and motion onset (Compare with Fig 2a in the main text). On the contrary, setting the threshold value to 30% maintains this relation but produces biased late detection events that skew the entire distribution to the right (see the two additional parallel lines at Fig S7b, and Compare with Fig 2a in the main text). Thus, setting the threshold value to 20% rise in the decay time of the autocorrelation balances well between false early detections and missing the rise in the decay time signal.

S5 Finger dot motion does not show early warning signals

In Experiment 2, participants responded to videos from two conditions - one shows an Attacker performing the task, the second shows a dot moving on the screen, tracking precisely the finger motion of the Attacker as seen on screen. Participants who saw the human Attacker responded 116 ms faster than the dot condition. Here we show that the dot trajectory does not show the early warning signals seen in the Attackers' center of mass motion, and thus strengthen the connection between Blockers' fast response times and the existence of the early warning signals.

To analyze the dot motion trajectory, we calculated the AR(1) and correlation decay time of the dot's motion trajectories. We find that for the AR(1) signal, there is no transition from negative to positive values, as observed for Attackers' center of mass motion (Fig S8a). Rather, for most of the trials, a positive autocorrelation is observed from the beginning of the trial (% positive initial AR(1) values in the first 100 ms of the trial = 87%, 95% CI =[86%,89%]). In line with the finding of the early positive autocorrelation, the autocorrelation decay time increase happened early in the beginning of the trial (Fig S8b). Not surprisingly, for most of the trials a sharp rise in the autocorrelation decay time was not a signal for motion onset (% of a 20% increase in the decay time in the first 200 ms of the trial = 71%, 95% CI =[69%,74%]).

To test whether the lack of early warning signals might be a consequence of the sampling of dot motion from Attackers' finger motion, we further analyzed the Attackers' finger motion in Experiment 1 (see main text for more details). We found that Attackers' finger motion did not show early warning signals. Similar to the dot motion analyzed above, its AR(1) signal started off positive and thus the decay time of the autocorrelation produced a sharp rise in the beginning of each trial. We repeated the same analysis as with the Attackers' center of mass motion (see main text), to find that there is no significant correlation between Attackers' motion onset and the rise in AR(1) or

with the autocorrelation decay rate (AR(1) signal: linear regression slope $\pm$ se $= 0.06 \pm 0.06$, p=0.35, Autocorrelation decay time: linear regression slope $\pm$ se $= -0.04 \pm 0.03$, p=0.23).

S6 The divergence of motion variance near the critical transition point is of a fold-bifurcation type

In this section we describe the fitting procedure of variance divergence near the transition point. For each trajectory we located the point of variance divergence (when the trajectory crosses the assigned threshold). We take 100 ms before and after this point and sync all trials in a block to the point of divergence (30 trials for each block). We averaged all trials in the block, resulting in one averaged trajectory per experimental block. For each mean trajectory, we locate the peak of the trajectory and take all the points distanced 40 ms from the start of the trajectory up to the peak in the averaged variance trajectory.

For each trajectory we choose a time-window of size 48 ms (12 data-points, see next section for fitting results for smaller time-windows). We partitioned each curve by the time window size, with an offset of 4 ms (1 data point). For each of the time window partitions we fitted the data with a non-linear model $log(\sigma^2) = -log(b) + nlog(t^* - t)$, where b, n, and t^* are fitting parameters of the model. We then chose the fitting parameters that provided the best fit across all time windows of each curve, resulting in 85 measures. We note that some of the curves were too noisy and the fitting procedure yielded noisy parameters. In Fig S9 we present the fitting curves of the first 35 variance curves.

S7 Variance divergence power-law is independent of the window size

In the main text, we showed that the distribution of power-laws from fitting the variance divergence near the transition point with a 48 ms time window suggests that variance divergence scales like $\sigma^2 \sim (t^* - t)^{-1/2}$. In Fig S10 we show that the distributions of power-laws for smaller time windows of 32 and 40 ms are similar and indicate the same power-law divergence of $n = -\frac{1}{2}$. In turn, this power-law suggests that the motor decision to move is of a fold-bifurcation type.

S8 Simulations of fold-bifurcation critical transition

In this section we describe the construction of the simulations of the fold-bifurcation critical transition events and their uncorrelated counterparts. To

test whether people can recognize a fold-bifurcation critical transition event we simulated a fold-bifurcation dynamical system. The fold-bifurcation system equations are:

$$\dot{x} = y + ax^2 - x^3 + \gamma\xi(t) \quad \text{(S1)}$$

$$\dot{y} = \epsilon \quad \text{(S2)}$$

The parameters of the model were chosen to be: $a = 4$, $\epsilon = 0.001$, $\gamma = 0.063$. We used Mathematica 11.1 ItoProcess function to simulate the Wiener process 100 times to create 100 trajectories. To remove the small bias in the motion before the transition point (and thus keep motion mean at the origin before the critical transition), we de-trended each trajectory before the transition and got 100 motion trajectories with a zero average before the transition. To randomize the direction of dot's motion we multiplied each trajectory by a random sign (± 1). The dot position on the screen is thus taken to be the x value of the fold-bifurcation motion trajectories (Fig S11a).

To compare the reaction time of people to the fold bifurcation events with an uncorrelated version of a transition, we created 100 trajectories which move from zero to the same final position, but instead of the correlated noise characterizing the critical transition, we added to the motion trajectories uniform random noise with a standard deviation set by the average standard deviation of the simulated fold-bifurcation trajectories described above. To randomize the direction of dot's motion we multiplied each trajectory by a random sign. Thus, we constructed 100 motion trajectories that show a similar transition from zero to a different location, but with uncorrelated noise which does not contain the early warning signals embedded in the fold-bifurcation transition (Fig S11b).

S9 Individual response times show a significant difference between the fold-bifurcation and uncorrelated noise motion simulations

In the main text we showed that averaging across participants, we get a significant faster response times for the fold-bifurcation dynamics compared with their uncorrelated counterpart dynamics (see previous section). In Table S1, we show that also comparing within-participants the response times across the 100 trials results in significant faster response times for the fold-bifurcation dynamics compared with their uncorrelated counterpart dynamics.

S10 Screening of outliers in the relation of motion onset to the early warning signals

In this section, we describe the screening procedure of outliers from the data of Attackers' and Blockers' motion onset. We calculated the relation between

motion onset of Attackers and Blockers and the early warning signals (a total of 2550 trials) by using linear regression. Plotting the relation between Attackers' and Blockers' motion onset and the early warning signals shows a clear correlation line (Fig S13a-d). Yet, in the extraction of the early warning signals there are false positive events that identify a rise in the autocorrelation decay time well before the critical transition occurs, which could incur an inflated value for the time by which the early warning signals precede motion onset. To exclude these outliers we used two screening procedures - First, we excluded all early warning signals that were earlier than 200 ms from the start of the trial. For the 40 ms time window, this procedure leaves 1756 trials for analysis. Second, we used these trials to calculate a density function and chose all trials that were in the region of density above a threshold. We chose 3 thresholds values: 1, 6, and 10 (for the 40ms time window). These thresholds result in 96%, 81%, and 64% of the data points respectively (see Fig S13b-d). The remaining data points were used for the calculation of the linear regression. We used the same procedure for all calculations of the lag time between the early warning signals (rise in the AR(1) signal and the rise in variance) and the Attackers' and Blockers' motion onset across all time window sizes. In the main text we report the results of the relation between Attacker's motion onset and the early warning signals for the density threshold value set to 6. The average time difference between Attacker's motion onset and the early warning signals when the threshold is set to 1 are: AR(1) average time difference of 147ms (Δt: mean $\pm$ ste $=147 \pm 2$ ms, $p < 10^{-5}$). Autocorrelation decay time time difference of 149ms (Δt: mean $\pm$ ste $=149 \pm 2$ ms, $p < 10^{-5}$). The average time difference between Blocker's motion onset and the early warning signals when the threshold is set to 1 are: AR(1) average time difference of 347ms (Δt: mean $\pm$ ste $=347 \pm 63$ ms, $p < 10^{-5}$). Autocorrelation decay-time, time difference of 329ms (Δt: mean $\pm$ ste $=329 \pm 45$ ms, $p < 10^{-5}$). The average time difference between Attacker's motion onset and the early warning signals when the threshold is set to 10 are: AR(1) average time difference of 128ms (Δt: mean $\pm$ ste $=128 \pm 2$ ms, $p < 10^{-5}$). Autocorrelation decay time time difference of 103ms (Δt: mean $\pm$ ste $=103 \pm 1$ ms, $p < 10^{-5}$). The average time difference between Blocker's motion onset and the early warning signals when the threshold is set to 10 are: AR(1) average time difference of 291ms (Δt: mean $\pm$ ste $=291 \pm 2$ ms, $p < 10^{-5}$). Autocorrelation decay-time, time difference of 278ms (Δt: mean $\pm$ ste $=278 \pm 1$ ms, $p < 10^{-5}$).

SI Figures

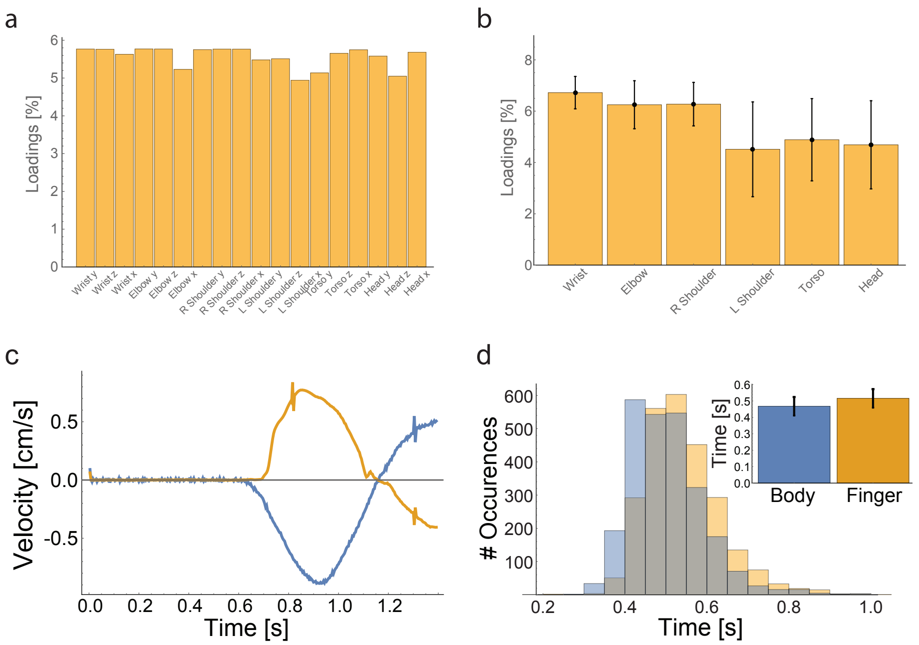

Figure S1: Attacker first PC of body motion is its center of mass which precedes finger motion. **a)** Example of the loadings of the first PC of Attacker body motion. **b)** Histograms of the averaged loadings of the six sensors. **c)** Motion of the Attacker's center of mass precedes motion of the Attacker's finger. Example of Attacker's body center of mass velocity (blue line) and Attacker's finger velocity (orange line). The two velocity traces are flipped in sign for a better view of their differences. **d)** Histograms of Attacker's center of mass motion onset (blue) compared to Attacker's finger motion onset (orange). **Inset**, Attacker's center of mass motion onset (blue) precedes finger motion onset (orange) by 52ms, $p < 10^{-5}$. Shown are mean±std.

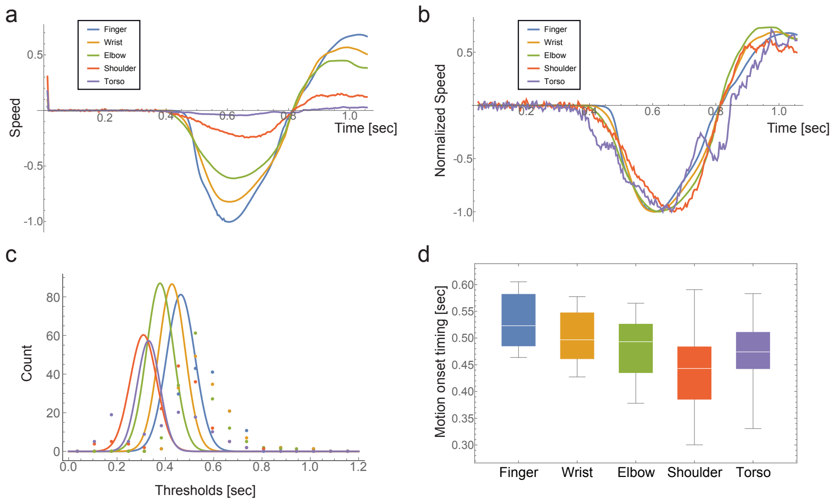

Figure S2: Motion of the Attacker's center of mass precedes motion of the Attacker's finger. **a)** An example of motion velocity of finger, whirst, elbow, shoulder, and torso sensors for Attacker's motion. **b)** Motion velocities as in a), normalized to their maximal value. **c)** An example of the Gaussian fits across sensors for a specific Attacker. Each distribution of motion onset per sensor was fit to a Gaussian and the mean and variance of the Gaussian distribution was extracted for each Attacker. **d)** The distribution of motion onset times for each body part across all Attackers. Line represents the median value, box boundaries the 25%-75% quantiles, and end lines the Min and Max valuse of the distribution. Median timing for motion onset and 50% CI: Finger: 523 ms, 50% CI=[485,582] ms, Wrist: 497 ms, 50% CI=[461,547] ms, Elbow: 493 ms, 50% CI=[435,526] ms, Shoulder: 443 ms, 50% CI=[385,484] ms, Torso: 474 ms, 50% CI=[442,510] ms.

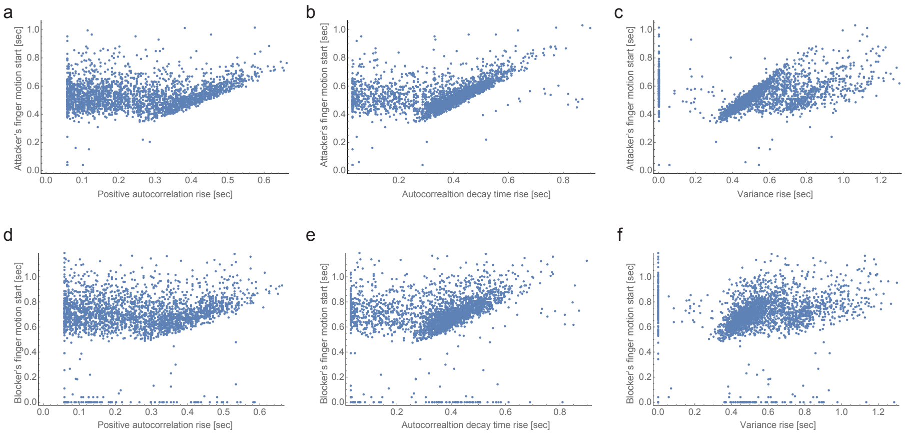

Figure S3: **Attacker's and Blocker's motion onset correlate with the early warning signals. a-c)** Attacker's finger motion onset as a function of a) The rise of the AR(1) signal, b) The increase in the autocorrelation decay time, and c) The rise of variance. For the linear regression calculations false positive events of AR(1), autocorrelation decay time, and variance, earlier than 200 ms from the beginning of the trial were removed. **d-f)** Blocker's finger motion onset as a function of d) The rise of the AR(1) signal, e) The increase in the autocorrelation decay time, and f) The rise of variance. For the linear regression calculations false positive events of AR(1), autocorrelation decay time, and variance, earlier than 200 ms from the beginning of the trial were removed.

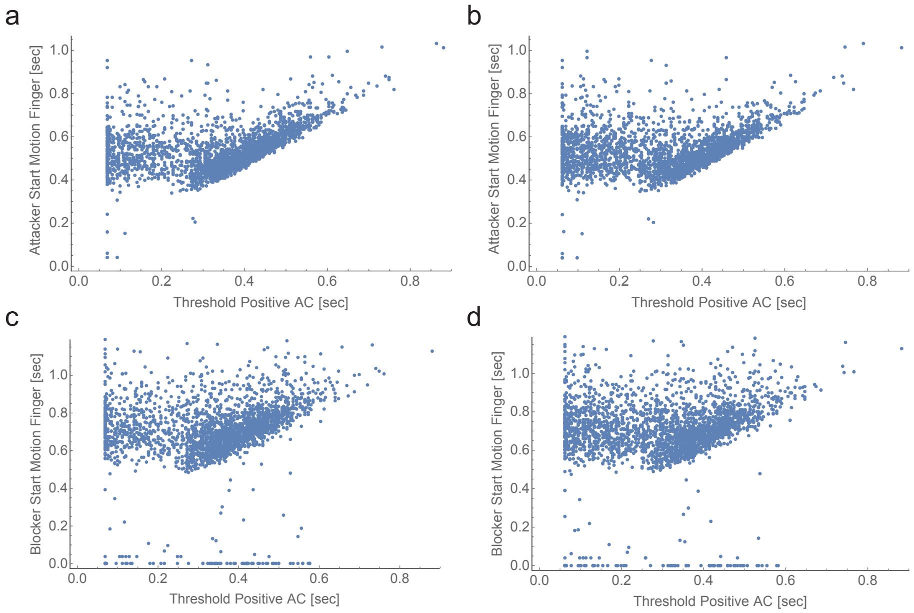

Figure S4: Autocorrelation at lag-1 sharp increase correlates with Attacker's and Blocker's finger motion onset also at time windows of 48 ms and 60 ms. **a)** Attacker's finger motion onset as a function of the rise of the AR(1) signal with a time window of 60ms. Time difference, Δt: mean $\pm$ ste $=132 \pm 1$ ms, $p < 10^{-5}$. **b)** Attacker's finger motion onset as a function of the rise of the AR(1) signal with a time window of 48ms. Time difference, Δt: mean $\pm$ ste $= 137 \pm 2$ ms, $p < 10^{-5}$. **c)** Blocker's finger motion onset as a function of the rise of the AR(1) signal with a time window of 60ms. Time difference, Δt: mean $\pm$ ste $=357 \pm 47$ ms, $p < 10^{-5}$. **d)** Blocker's finger motion onset as a function of the rise of the AR(1) signal with a time window of 48ms. Time difference, Δt: mean $\pm$ ste $= 388 \pm 56$ ms, $p < 10^{-5}$.

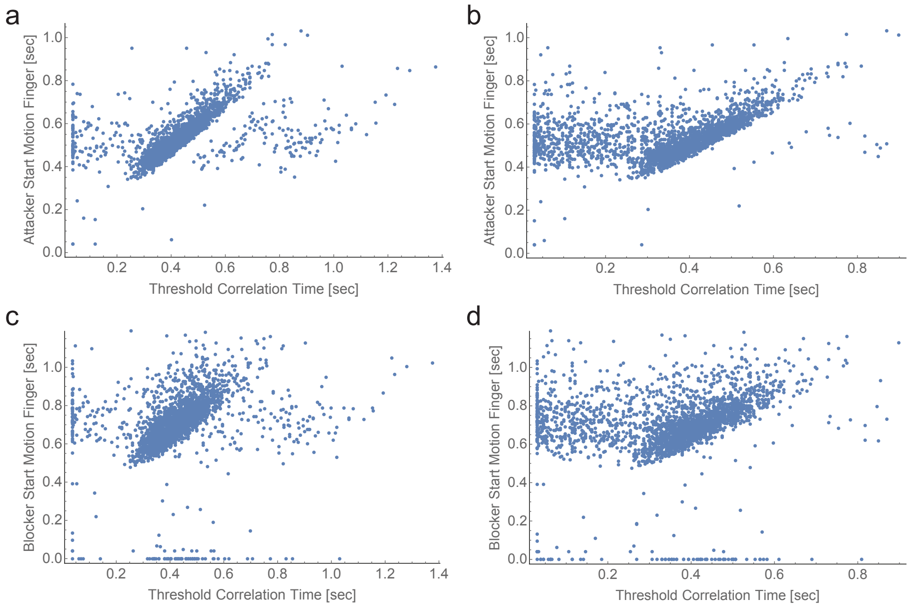

Figure S5: Autocorrelation decay time sharp increase correlates with Attacker's and Blocker's finger motion onset also at time windows of 48 ms and 60 ms. **a)** Attacker's finger motion onset as a function of the rise of the autocorrelation decay time with a time window of 60ms. Time difference, Δt: mean $\pm$ ste $=107 \pm 1$ ms, $p < 10^{-5}$. **b)** Attacker's finger motion onset as a function of the rise of the autocorrelation decay time with a time window of 48ms. Time difference, Δt: mean $\pm$ ste $= 126 \pm 1$ ms, $p < 10^{-5}$. **c)** Blocker's finger motion onset as a function of the rise of the autocorrelation decay time with a time window of 60ms. Time difference, Δt: mean $\pm$ ste $=90 \pm 27$ ms, $p = 0.001$. **d)** Blocker's finger motion onset as a function of the rise of the autocorrelation decay time with a time window of 48ms. Time difference, Δt: mean $\pm$ ste $= 320 \pm 37$ ms, $p < 10^{-5}$.

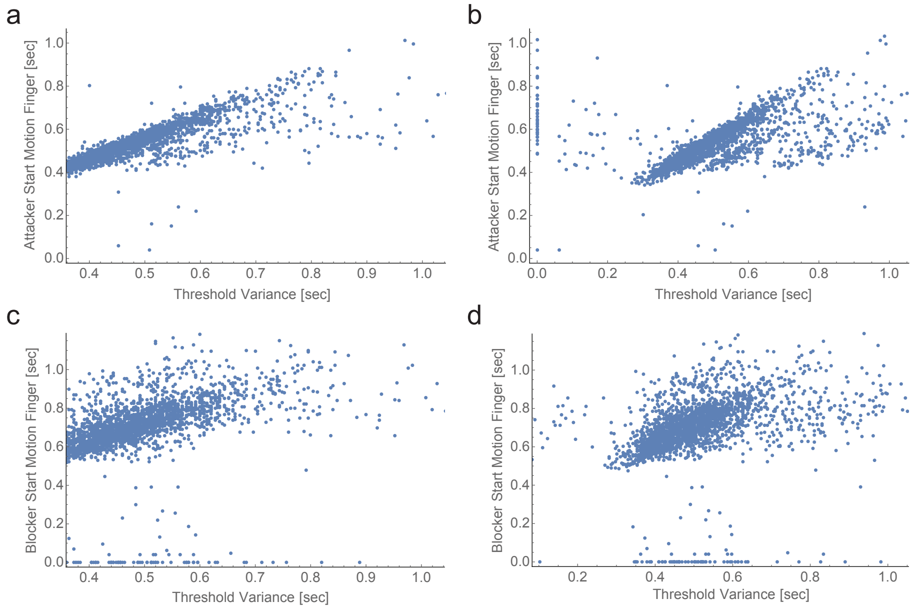

Figure S6: Variance sharp increase correlates with Attacker's and Blocker's finger motion onset also at time windows of 48 ms and 60 ms. **a)** Attacker's finger motion onset as a function of the rise of the variance with a time window of 60ms. Time difference, Δt: mean $\pm$ ste $=50 \pm 1$ ms, $p < 10^{-5}$. **b)** Attacker's finger motion onset as a function of the rise of variance with a time window of 48ms. Time difference, Δt: mean $\pm$ ste $= 23 \pm 2$ ms, $p < 10^{-5}$. **c)** Blocker's finger motion onset as a function of the rise of the variance with a time window of 60ms. Time difference, Δt: mean $\pm$ ste $= 234 \pm 1$ ms, $p < 10^{-5}$. **d)** Blocker's finger motion onset as a function of the rise of the variance with a time window of 48ms. Time difference, Δt: mean $\pm$ ste $= 215 \pm 2$ ms, $p < 10^{-5}$.

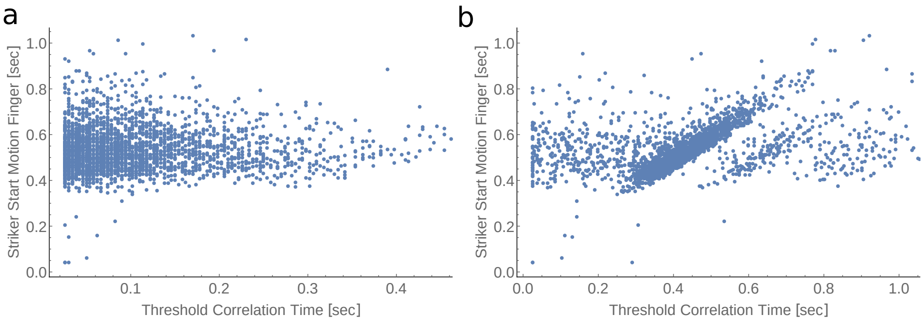

Figure S7: **Autocorrelation decay time threshold balances accuarate detection and false positives. a)** A threshold value of 10% rise in the autocorrelation decay time as the threshold for the sharp rise in the autocorrelation decay time shows a high rate of false positives. Shown is Attacker's finger motion onset as a function of the rise of the autocorrelation decay time with a time window of 40ms and a threshold value for the autocorrelation decay time rise of 10%. Compare with Fig 2a in the main text. **b)** A threshold value of 30% rise in the autocorrelation decay time as the threshold for the sharp rise in the autocorrelation decay time shows a late detection of the rise in the autocorrelation decay time. Shown is Attacker's finger motion onset as a function of the rise of the autocorrelation decay time with a time window of 40ms and a threshold value for the autocorrelation decay time rise of 30%. Compare with Fig 2a in the main text.

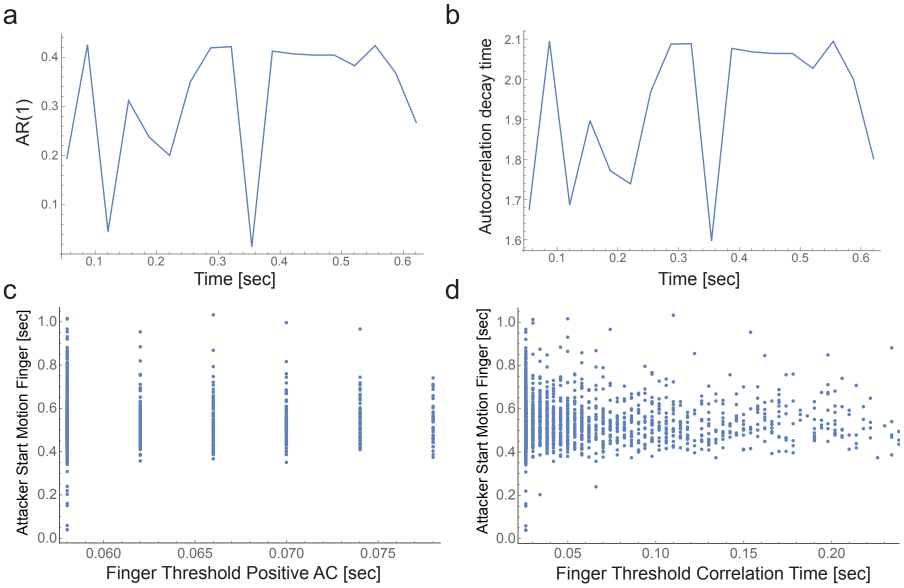

Figure S8: **Finger dot motion does not show early warning signals. a)** A sample trace of the autocorrelation at lag-1 signal (AR(1)) of a dot motion trajectory from Experiment 2. The AR(1) starts positive for most of the dot motion trajectories (% positive initial AR(1) values in the first 100 ms of the trial = 87%, 95% CI =[86%,89%]). **b)** A sample trace of the autocorrelation decay time of a dot motion trajectory from Experiment 2. The autocorrelation decay time has a sharp rise at the beginning of the trial for most of the dot motion trajectories (% of a 20% increase in the decay time in the first 200 ms of the trial = 71%, 95% CI =[69%,74%]). **c)** Analysis of finger motion in Experiment 1 shows that there is no significant correlation between Attackers' motion onset and the first timing of positive values for the finger's AR(1) signal (linear regression slope $\pm$ se = 0.06$\pm$0.06, p=0.35). **d)** Analysis of finger motion in Experiment 1 shows that there is no significant correlation between Attackers' motion onset and the sharp increase of the finger's autocorrelation decay time (linear regression slope $\pm$ se = -0.04 ± 0.03, p=0.23).

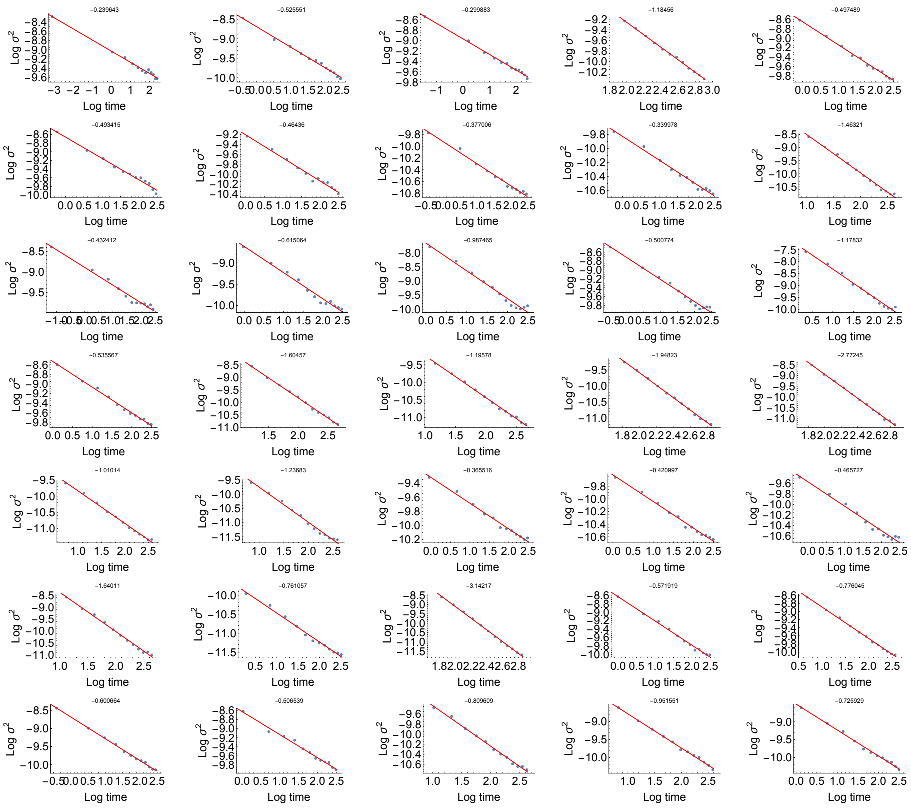

Figure S9: Variance sharp rise near the transition point shows a fold-bifurcation type of divergence.

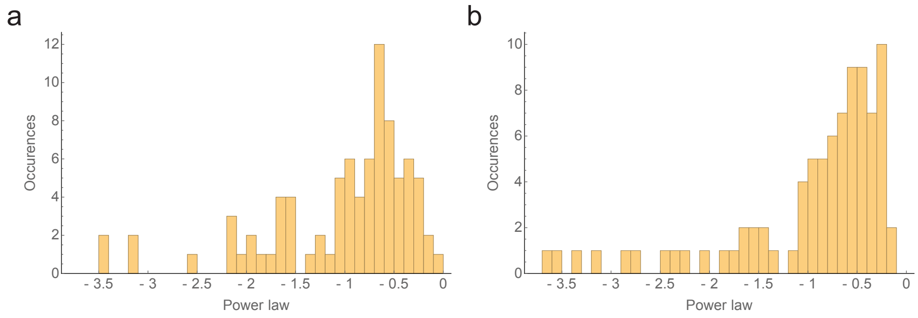

Figure S10: **scaling power-law for different window sizes shows a divergence with power law of -0.5. a)** Histogram of extracted power-laws using a window size of 32 ms in the fitting procedure. Median power-law=-0.65, 95% CI =[-0.70,-0.55]. **b)** Histogram of extracted power-laws using a window size of 40 ms in the fitting procedure. Median power-law=-0.58, 95% CI =[-0.66,-0.49].

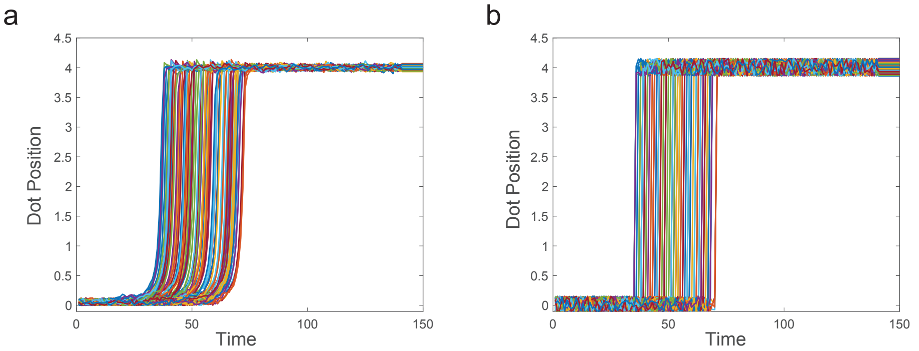

Figure S11: **Trajectory samples for the fold-bifurcation simulations and their uncorrelated counterpart. a)** Trajectories of the fold bifurcation dynamics (before the randomization of their direction). The parameters of the model were chosen to be: $a = 4$, $\epsilon = 0.001$, $\gamma = 0.063$. **b)** Trajectory examples for the uncorrelated version of the transition. To a unit-step transition between zero to 4, we added uniform random noise. Transition points of the uncorrelated simulations were taken to match the transition points of the fold-bifurcation critical transition events.

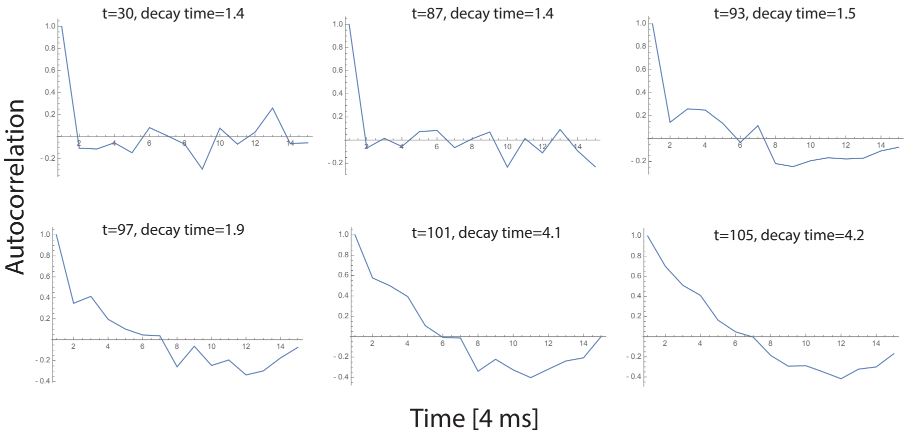

Figure S12: **As the transition point approaches, the autocorrelation decay time rises sharply.** An example for the sharp increase in autocorrelation decay time before the transition point. Time points for the autocorrelation calculation were (in ms): 30, 87, 91, 97, 101, and 105 ms from the trial's beginning. Indicated are the corresponding autocorrelation decay time (in time points units, 4 ms apart): 1.4, 1.4, 1.5, 1.9, 4.1, 4.2. In the main text, the time point at which the decay time shows a 20% increase compared to its previous value is marked as the time point of the sharp rise in the autocorrelation decay time.

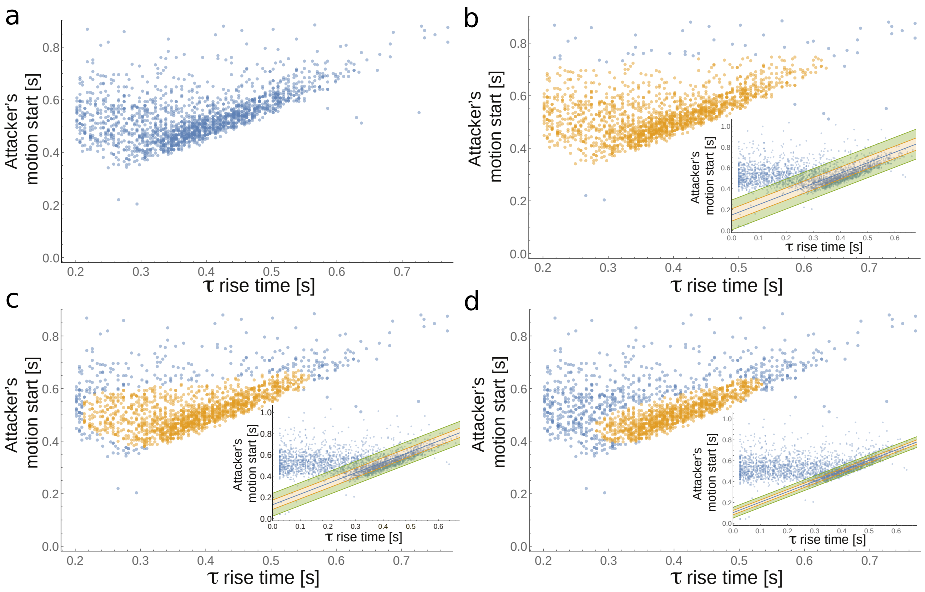

Figure S13: **The different density thresholds taken to screen outliers in the Attackers' relation with the early warning signals. a)** Data points before screening of outliers for Attacker's motion onset versus the rise in the autocorrelation decay time with a time window of 40ms (see Fig 2a, main text). This and all other plots exclude all data points stemming from early warning signals that preceded 200 ms from trial start time **b)** Included data points with a density threshold contour of 1 or higher (marked in orange, 96% of all data points). **Inset**, The linear regression line (solid blue), 50% CI (solid oragne) and 90% CI (solid green) lines. Δt: mean $\pm$ ste $=147 \pm 2$ ms. **c)** Included data points with a density threshold contour of 6 or higher(marked in orange, 81% of all data points). **Inset**, The linear regression line (solid blue), 50% CI (solid oragne) and 90% CI (solid green) lines. Δt: mean $\pm$ ste $=131 \pm 2$ ms. **d)** Included data points with a density threshold contour of 10 or higher (marked in orange, 60% of all data points). **Inset**, The linear regression line (solid blue), 50% CI (solid oragne) and 90% CI (solid green) lines. Δt: mean $\pm$ ste $=103 \pm 1$ ms.

SI Tables

Table S1: Response times (mean±ste) for fold-bifurcation and uncorrelated motion simulations per participant

Participant	Fold bifurcation	Uncorrelated	p-value
1	1.16 ± 0.002	1.23 ± 0.002	0.01
2	1.17 ± 0.002	1.29 ± 0.002	10^{-5}
3	1.08 ± 0.002	1.20 ± 0.002	$2 * 10^{-4}$
4	1.12 ± 0.002	1.29 ± 0.002	10^{-8}
5	1.04 ± 0.002	1.17 ± 0.002	10^{-6}
6	1.12 ± 0.002	1.20 ± 0.002	0.001
7	1.12 ± 0.002	1.22 ± 0.002	0.001
8	1.21 ± 0.002	1.25 ± 0.002	0.054
9	1.23 ± 0.002	1.28 ± 0.002	0.043
10	1.06 ± 0.002	1.18 ± 0.002	10^{-5}
11	1.19 ± 0.002	1.25 ± 0.002	0.014
12	1.26 ± 0.002	1.37 ± 0.002	$2 * 10^{-4}$
