# Supplementary material for: Early warning signals in motion inference
Source: PLoS Comput Biol. 2020 May 29;16(5):e1007821. doi: 10.1371/journal.pcbi.1007821 (PMC7259514; doi:10.1371/journal.pcbi.1007821)
Supplement: Attachment — Submitted filename: CoverLetter_PCB.pdf [file pcbi.1007821.s018.pdf]

HARVARD UNIVERSITY
School of Engineering and Applied Sciences
Center for Brain Science
Department of Physics
Pierce Hall 324, 29 Oxford Street, Cambridge, MA 02138, USA
WEB: http://www.seas.harvard.edu/softmat

L. MAHADEVAN
Lola England Professor of Applied Mathematics
Professor of Organismic and Evolutionary Biology
Professor of Physics
tel: (617) 496-9599 ; fax: (617) 495-9837
email: lmahadev@g.harvard.edu

March 1 2020

Editor, PLoS Computational Biology

Dear Editor,

We would like to submit a revision of our manuscript titled: “Early warning signals in motion inference” for consideration in PLoS Computational Biology.

We thank the reviewers for their help in improving the manuscript and revised the manuscript according to their comments. The revised manuscript has a more inclusive discussion of the wider joint-action and social motor coordination literature, it is more rigorous in its data visualization, and clearer in its notations and equations. Please see a point-by-point reply to reviewers’ comments below.

Thank you for your consideration,

Yours sincerely,
L. Mahadevan

Reviewer #1: The manuscript details three experiments and a set of simulations exploring the subtle movement dynamics that human actors are sensitive to when anticipating or predicting another actor's movement intention. Using an actor-defender stimulus-response paradigm, the results of empirical experimentation and simulation work revealed that defending actors are sensitive to early warning signals in an actor's center of mass (or principle component motion time-series) that occur approx. 150 ms before the onset of an actor's task specific movement. Moreover, the dynamics of an attacker's motion near the point of movement transition corresponds to the dynamics of a fold-bifurcation, a fundamental property of nonlinear dynamical systems.

This is a fantastic piece of work. The experimental paradigms and stimuli employed, although simply, and well-conceived and provide a valid method of determining the subtle movement dynamics that help observers (defenders) anticipate or infer the action intention of an observed opponent. The data analysis and modelling techniques are highly novel with respect to the phenomena investigated, with the overall findings likely to have a transformative impact with the fields of human-movement science, perception-action and joint-action and social motor control and coordination. The writing is excellent, with the method, figures and supplementary material well-constructed and easy to follow.

We thank the reviewer for this comment.

My only concern relates to the discussion section, which is largely just a summary of the findings, and does not effectively situate the work within the wider joint-action and social (multiagent) motor coordination and action observation literature. Research on action observation and cooperative and competitive performance has a long history in psychology, cognitive science and human-movement science, with a particular increase in focus over the last two decades. A core debate within this literature, is whether the a purely neuro-cognitive or representational approach (e.g., Prinz, Sebanz and Knobloch and colleagues co-representational/motor resonance account of joint action and action observation; as well as the work on mirror neurons and motor simulation by numerous neuroscientists and cognitive neuroscientists) is required to explain such phenomena in comparison to a more ecological-dynamical (informational) account (e.g., Araujo Duarte and colleagues, Christina Becchio and colleagues, Schmidt and Richardson and colleagues; Yuji Yamamoto and colleagues, in addition to Kelso and colleagues). While these two general approaches to action observation and coordination are perhaps best understood as complementary, the current work is highly relevant to these theoretical issues and the work might have more general impact if the authors better situate this work within the context

of this wider literature. Note, that I am not saying that the authors need to make any strong theoretical claims, or even articulate any theoretical conclusions one way or the other, but rather better detail how the work relates to the previous work on the dynamics of action observation and prediction that exists within the joint-action and social motor control literature.

I appreciate that the authors would probably like a list of key references, but this literature is rather large and I do not wish to bias the authors assessment of this literature or what they feel should or should not be included. The authors should also see the above listed names as a rather limited set of relevant researchers and there are many more that could be considered.

We thank the reviewer for this helpful comment. In the revised MS, we added a new paragraph that situates our work in the wider scope of joint-action and social motor control literature with a specific reference to the differences between the co-representational approach and the informational approach. In the Discussion section, page 12, line 391 we added:

“Our work explored the informational cues that are inferred by the Blocker from the Attacker's motion. It draws on a cohort of previous works on the dynamical aspects of joint-action and social motor coordination [30, 39-41]. For example, participants react differently to grasping movements depending on the social cues they infer from their partner's motion [42, 43] and team sports players move according to the affordances incurred by their teammates and their opponents [44, 45]. To complement this view, further study of possible co-representations in Attacker and Blocker's motoric and neuronal activity can indicate whether motor simulation [7, 46, 47] plays a role in these interactions and elucidate the biological mechanisms that may underlie such accurate inference of the other's intention [48-52].”

Reviewer #2:

## Summary of the article

The article addresses an interesting paradox brought by recent studies in motor behaviour: people are able to predict others' motion trajectories even before the start of their movement. The authors used a simple and elegant paradigm called the "antagonistic game" where one player —the Blocker— has to parry on the movement of another player –the Attacker. In a first experiment, they demonstrated how the fluctuations of the centre of mass of the Attacker's body are actually giving early signs of his/her ensuing movement 150ms before it starts. The authors then demonstrate how those fluctuations show characteristics of a saddle-node bifurcation. And finally, simulating similar dynamics and presenting them to human participants, they show how the simulated movements also induce faster reaction times.

## Main impressions of the article

The study is very interesting and, despite its technical aspects, remains concise and clearly written. I think it has the sufficient impact for PLoS Computational Biology ensuing few minor corrections.

We thank the reviewer.

## Comments and suggestions

Note: it would have been nice to use a one column presentation, and, to add page and line numbers to ease the review.

Following the reviewer's suggestion, we changed the format of the manuscript to a single column format and added page and line numbers.

Title, Abstract, and Introduction are well written and the former covers well existing literature.

We thank the reviewer.

In the autocorrelation formula, describes what is "arg_n".

Following the reviewer's comment we added on page 6, line 200:

"and arg_n chooses the time scale n that holds the equation , and thus measures the time scale for the autocorrelation to decay to of its maximal value."

To what extent the different measures of autocorrelation and variance across time window is modulated by sampling frequency?

Following the reviewer’s comment, we clarify in the revised manuscript in the Methods section, Experiment 1, line 78 the following: “For accurate measures of the response times we used the maximal sampling frequency available. Changing the length of the time windows did not change the current results, see SI sections S3 and S7.”

The authors define "the rise of autocorrelation decay time" with a decrease of “20% between two consecutive time windows” but do not explicit why.

Following the reviewer’s comment, we added a new section to the SI (section S4 and Figure S7), discussing the reasons for setting the threshold for a rise in the autocorrelation decay time to a 20% change and presenting analysis results with other threshold values. In addition, in the revised manuscript on page 6, line 212 we added the following: “The 20% fold change threshold was set to filter out noisy detection events while maintaining accurate detection of a rise in the autocorrelation decay time (see SI, S4).”.

The 347ms time difference observed between the AR(1) sharp rise and Blockers' start time remind the P300 in the EEG literature. That may be interesting to discuss.

We thank the reviewer for this comment. In the revised MS, we added to the discussion on page 12, line 380: “We note that the observed 300ms time difference between the early warning signals and Blockers' response time might relate to the P300 signal in EEG literature [35, 36]. Further studies of our experimental paradigm for social interactions in EEG setting (similar to Refs [37, 38]) could indicate if that is indeed the case.”

Fig. 2: Add the linear regression line and confidence interval. Maybe the two panels can be combined using two colours, thus helping comparison between the two measures.

We thank the reviewer for this helpful comment. Following the reviewer’s suggestion, we added the regression line and confidence intervals to Fig 2. Joining the two panels together made the figure a bit too crowded so we decided to keep the two panels format. We also added to the Methods section and the SI, section S10, details on the linear regression and confidence intervals procedure and its dependence on different threshold values. We also report the resulting linear regression fitting parameter as a function of this data inclusion threshold. We also revised Fig S13 to demonstrate the fitting results and their CI for different choices of the threshold values for data points inclusion in the fitting process.

This is not clear why the AR(1) signal of Attacker's finger starting positive implicates that decay time only makes a sharp rise at the beginning of each trial.

Following the reviewer’s comment, in the revised manuscript we rephrased this sentence to make it clearer. On page 8, line 268 we added (marked in red): “We found that finger motion of the Attacker did not show early warning signals; Its AR(1) signal started off positive and thus the decay time of the autocorrelation starts at high values from the beginning of the trial and does not rise further. Analysis of Attackers' finger motion indicates no significant correlation between Attackers' motion onset and the rise in AR(1) or in the autocorrelation decay rate of finger motion.”.

Would it be possible to briefly explain how (3) is derived from (1-2)?

Following the reviewer’s comment, in the revised manuscript, page 9, line 302, we clarify the derivation of Equation 3 (Eq. 5 in the revised manuscript) :

“The inverse square root relation between the variance and the time interval to transition reflects the system’s fluctuations as it reaches the departure point from the stable manifold on which the scaling relations are: x~ (Eq (3)) and y~t (Eq (4)) [22,23].”

Fig. 4: Getting a sense of distributions is important. Violin plot or even the recent raincloud plots would be perfect here.

We thank the reviewer for this comment. In the revised manuscript we changed Fig 4 to be in a Violin plot format.

Adding videos of the two types of simulated dynamics would be great to get a subjective sense of the difference.

Following the reviewer’s comment we added two videos of the simulated dynamics in the fold-bifurcation case and its uncorrelated counterpart.

This is not clear why the increase of autocorrelation decay time is less noisy than the AR(1)

Following the reviewer’s comment, in the revised manuscript, Discussion section, page 11, line 375, we clarify the following: “The reaction by Blockers to early warning signals raises the question of the underlying mechanism that governs this early detection ability. Our data analysis process indicated that the detection of the increase in the autocorrelation decay time was less affected by noisy measurements than the AR(1) signal (See Fig 3, and compare Figs S2-S4 in the SI). Computationally, it might suggest a preference for the measurement of the decay time of the autocorrelation as a cue for Blockers' action.”

This is not clear why AR(1) would necessarily need to store in memory two timeseries (cannot be implemented through dynamical systems without a

memory per see). Is there a formal proof and a reference supporting this claim?

We thank the reviewer for this comment. We removed the sentence suggesting that it is necessary to hold in memory two time series in order to calculate the AR(1) signal.

In the method, this is not clear how the dot projected on a 2D screen can mimic human dynamics which is in 3D.

Following the reviewer's comment, we clarify in the Methods section of the revised manuscript that the 2D dot motion is a toy model of the 3D motion. In the Methods, Experiment 3, page 4, line 118 we write: "We note that the 2D motion of the dot cannot mimic fully the 3D motion of people but rather serves as a proxy to check whether people are sensitive to the difference between a fold-bifurcation transition with its correlated noise and an uncorrelated counterpart."

How the authors have chosen the parameters of the dynamical systems (alpha & gamma)? Does the choice of those values strongly impact the results?

Following the reviewer's comment we clarify in the revised manuscript, Methods section, Experiment 3, page 4, line 115: "We chose the values of the and parameters, as well as the frame rate and task duration in the simulations to capture the same dynamics and transition time observed in the behavioral experiments of the dyadic Attacker-Blocker interactions.".
