# Supplementary material for: Early warning signals in motion inference
Source: PLoS Comput Biol. 2020 May 29;16(5):e1007821. doi: 10.1371/journal.pcbi.1007821 (PMC7259514; doi:10.1371/journal.pcbi.1007821)
Supplement: Attachment — Submitted filename: CoverLetter.pdf [file pcbi.1007821.s019.pdf]

HARVARD UNIVERSITY
School of Engineering and Applied Sciences
Center for Brain Science
Department of Physics
Pierce Hall 324, 29 Oxford Street, Cambridge, MA 02138, USA
WEB: http://www.seas.harvard.edu/softmat

L. MAHADEVAN
Lola England Professor of Applied Mathematics
Professor of Organismic and Evolutionary Biology
Professor of Physics
tel: (617) 496-9599 ; fax: (617) 495-9837
email: lmahadev@g.harvard.edu

March22, 2020

Editor, PLoS Computational Biology

Dear Editor,

We would like to submit the final version of the manuscript titled: “Early warning signals in motion inference” for consideration in PLoS Computational Biology.

In response to your query to show all the data, we have now included these in our revised Fig. 4 which takes a graphical form, and in addition have been cautious about using t-tests.

We have also modified Fig. 1 to show the task more clearly, and been more consistent with terminology throughout the paper, particularly in the use of the phrase “fold transition” rather than saddle-node bifurcation.

The final version of the MS along with the figures has now also been uploaded to the site. We look forward to seeing the proofs of the paper in a short time.

Thank you for your consideration,

Yours sincerely,
L. Mahadevan
